# Characterization of a 16-Year-Old Long-Time Survivor of Edwards Syndrome

**DOI:** 10.7759/cureus.15205

**Published:** 2021-05-24

**Authors:** Farheen Khan, Iqra Jafri

**Affiliations:** 1 Pediatrics, Dubai Hospital, Dubai, ARE; 2 Genetics, Pediatrics, Dubai Medical College, Dubai, ARE

**Keywords:** edwards syndrome, aneuploidy, pediatric genetics, survival rate, continuity of care

## Abstract

Edwards syndrome, or trisomy 18, is an uncommonly encountered aneuploidy in which multiple organs are affected and have compromised function. Only 13% of neonates born with Edwards syndrome survive beyond their first year of life. In this paper, we report the case of a 16-year-old girl with non-mosaic (with meiotic non-disjunction) Edwards syndrome who survived long beyond the expected life span of less than two years. She was diagnosed by karyotyping at the age of one month with complete trisomy 18. She had global developmental delay, a diaphragmatic hernia, recurrent chest infections, juvenile idiopathic scoliosis of the thoracolumbar region, neurogenic bladder, fecaloma, bilateral exposure keratopathy, and failure to thrive.

## Introduction

The incidence of Edwards Syndrome is 1/8,000 live births, although the prevalence is much higher at 1/2,600 pregnancies. This discrepancy can be accounted for by deaths in utero, pregnancy terminations, and fetal loss. There are different forms of the syndrome: mosaic, full, and Robertsonian translocations (partial). The mosaic and partial forms are milder than the full form, although a great variation in symptoms can be seen in all forms [1-4].

Along with Down syndrome, Edwards syndrome is screened for prenatally by assessing nuchal translucency on ultrasound and by prenatal blood tests. However, it is impossible to predict the severity of the effect of the syndrome on the child prenatally (even if diagnosed by amniocentesis or chorionic villus sampling). Often, the diagnosis of Edwards syndrome is not made prenatally due to lack of screening, diagnostic testing, or prenatal care. In these cases, the diagnosis can be made postnatally based on several criteria. These include intrauterine growth retardation, specific dysmorphic features, congenital heart malformation (particularly ventricular septal defects), characteristic extremity malformations such as rocker bottom feet and overlapping fingers, short sternum, and prominent occiput. All of these features may be present at birth but not all may occur in a single patient, and the features vary in severity. Other differential diagnoses for similar features at birth may include thrombocytopenia absent radius syndrome, Roberts syndrome, and Smith-Lemli-Opitz syndrome [3,5].

According to the National Down Syndrome Cytogenetic Register (UK), the median survival rate of infants with trisomy 18 is 14 days while the one-year survival rate is a mere 8%. Death in these children is mainly due to organ failure or major organ dysfunctions. Care for children living with Edwards syndrome is typically multidisciplinary and lifelong [5].

## Case presentation

The patient is a 16-year-old girl with Edwards syndrome which was diagnosed clinically at birth. The diagnosis was confirmed again by karyotyping seven years later.

The child was born to a 30-year-old, P3+0 mother at 37 weeks of gestation after an uneventful pregnancy and good maternal antenatal care. On antenatal scans, liquor was adequate and the fetus was identified as having intrauterine growth retardation. The birth was by a lower segment Caesarean section, with a birth weight of 1.6 kg and poor Apgar scores (6 at one minute and 8 at five minutes). Her mother had completed her immunizations, and all tests of the infant postnatally were negative for any infections. She did not breathe spontaneously at birth and did not cry until four minutes later. She was ventilated for two days at birth after failed nasal intubation due to unilateral choanal atresia. She was also given phototherapy for neonatal jaundice for seven days. She was kept in the neonatal intensive care unit for 35 days. During this time, on examination, a grade two ejection systolic murmur was heard, and an echocardiography revealed mild hypertrophic cardiomyopathy, which improved with time and caused no outflow obstruction. An ultrasound scan of the head on day one revealed a choroid plexus cyst. She passed meconium on day two, and chest and abdominal examinations were normal. She was initially kept nil per os and later switched to oral feeds because she was not sucking well initially. She was breastfed up to the age of six months, after which she continued on formula.

At one year of age, she failed the brainstem electric response audiometry screening test for hearing with varying low scores bilaterally. In her early years, she had global developmental delay and had multiple hospital admissions requiring intravenous antibiotics for episodes of broncho and lobar pneumonia.

**Figure 1:**
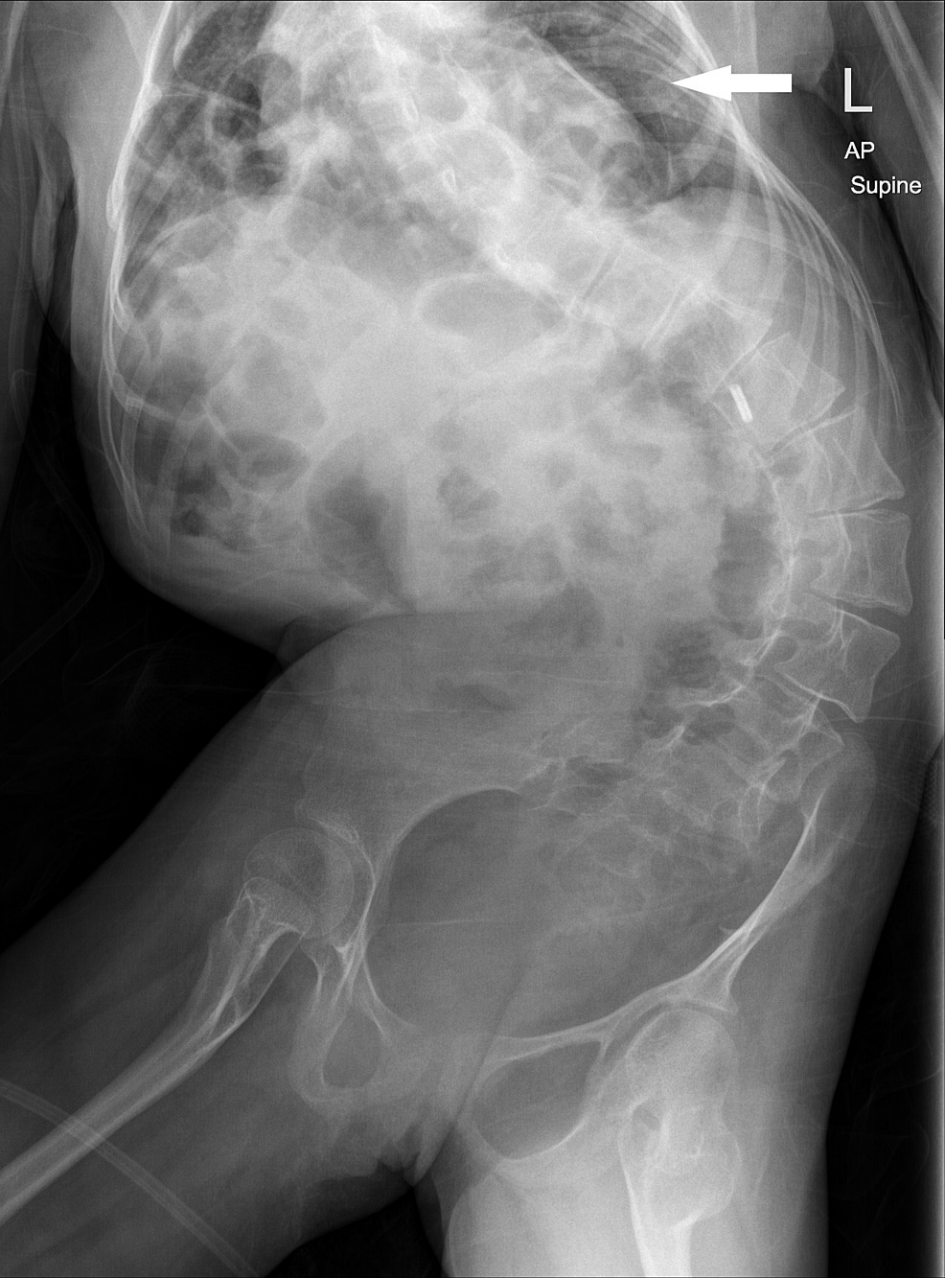
Chest and abdominal X-ray showing thoracolumbar scoliosis and left-sided diaphragmatic hernia with a hypoplastic lung (white arrow).

At the age of four, she was diagnosed with neurogenic bladder by a cystourethrogram which showed multiple bladder diverticula and associated trabeculation. The cystourethrogram was done due to the presence of a suprapubic mass and suspected right ureterocele but results suggested neurogenic bladder. She was also diagnosed with a Morgagni diaphragmatic hernia at four years of age when intestinal loops were seen on a chest X-ray done during a hospital admission due to an episode of bronchopneumonia (Figure 1). However, no surgical intervention was done and the intestinal loops currently persist in the thoracic cavity.

At two years of age, she was diagnosed with epilepsy. After taking the required course of phenobarbitone and being seizure-free for two years, she discontinued the medication.

On a routine visit to the ophthalmology clinic, she was diagnosed with exposure keratitis (at age 14) and was advised conservative treatment. She also has myopia and requires the use of glasses.

Currently, the patient attends regular follow-ups with the ear, nose, and throat (ENT) clinic, along with the pediatric ophthalmology clinic, and is often admitted to the hospital due to recurrent bronchopneumonia. Her most recent echocardiogram showed no abnormalities. She has spastic quadriplegia and contractures which require regular physiotherapy and the use of a special wheelchair. She has failure to thrive with growth parameters of height, weight, and head circumference well below the third centile and therefore requires regular monitoring. Her pubertal development is normal, with regular menses. She has no known allergies and is on a soft diet orally as the insertion of a gastrostomy tube was refused by the parents. In the neonatal period, she had trouble swallowing and sucking. The trouble with swallowing is ongoing and may be the cause of her recurrent aspiration pneumonia. She urinates and passes stool spontaneously but wears a diaper. She has had chronic constipation since the age of four and often requires the use of laxatives.

On inspection, she has the typical features of Edwards syndrome, namely, prominent occiput, short sternum, low set ears, low hairline, micrognathia, overlapping fingers, and rocker bottom feet.

**Figure 2:**
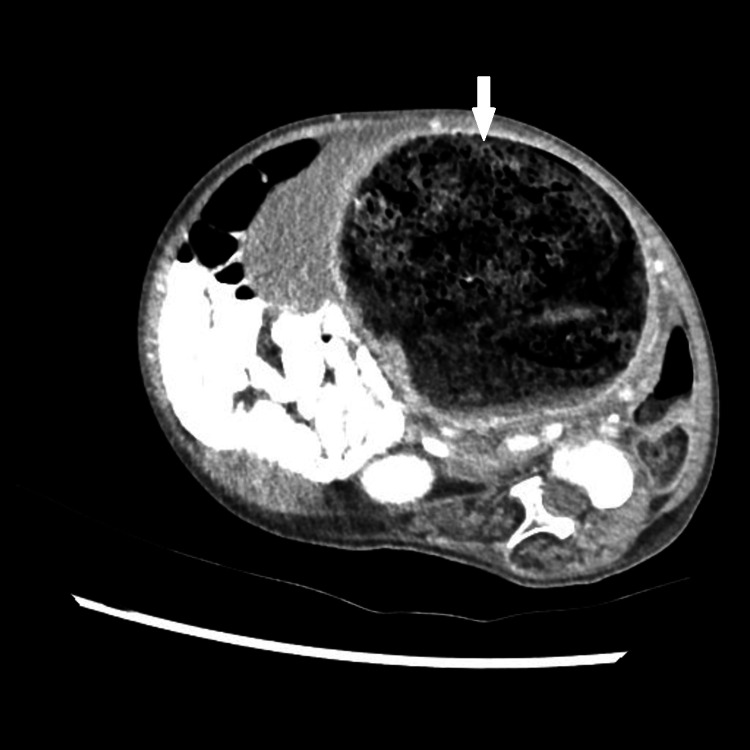
CT scan showing a large fecaloma (white arrowhead). CT: computed tomography

Her most recent admission to the hospital was due to fever, abdominal pain, and constipation for the past four days. She also had poor oral intake recently and had not been taking laxatives (Movicol). Computed tomography revealed a fecaloma reaching the intestinal loops posterior to the sternum (Figure 2). On examination, she was febrile with a hard, distended abdomen and significantly reduced bowel sounds. She was treated with fleet enema, ceftriaxone, and paracetamol and was discharged within a week.

She has no known family history of any genetic disorders, syndromes, or blood-related illnesses. Her parents are non-consanguineous and she has four healthy siblings.

## Discussion

Edwards syndrome presents with a multitude of symptoms affecting different organs. Therefore, it is crucial to maintain a multidisciplinary approach throughout the life of the patient. This helps ensure optimum quality of life. As we have seen, coordination was required between the pediatric neurologist, general pediatrician, ophthalmologist, ENT specialist, and physiotherapist to give the patient the best possible care. It is also necessary to understand that not all pathologies can be dealt with in a typical manner. For example, diaphragmatic hernia in any other child would have been corrected immediately. However, her thoracolumbar scoliosis and other comorbidities due to the syndromic effects prevented this and it remains uncorrected. A responsible pediatrician must have a high level of suspicion for common pathologies (i.e., heart defects) and look to prevent and treat them early as was done for her epilepsy. However, hypertrophic cardiomyopathy was an unusual finding with regard to the syndrome in her case. Continuous monitoring and support from both the family and physicians are necessary, particularly for chronic problems such as quadriplegia and failure to thrive.

The burden on the caregivers and other family members must also be kept in perspective as financial, physical, and emotional burdens are greater for parents of children with chronic illnesses [6]. In this case, the parents were offered a gastrostomy tube to be inserted in the patient; however, they preferred to continue oral feeding for her so as to not have her undergo any more treatments than necessary, despite making it more difficult for them to feed her. Her caregivers and family members are very supportive and are managing her care well and attending appointments and following up regularly along with care for their other children. This is extremely important to note in families of a child with a chronic illness as some studies have proven the increased incidence of mental health disorder in siblings of children with chronic illness requiring a high level of care. it must be ensured that these children are not neglected or emotionally deprived, without compromising the care of the ill child [7].

## Conclusions

It is quite rare to encounter a case similar to the one described here as few patients diagnosed with the full form of Edwards syndrome live to see their first birthday. This is due to the range of congenital malformations associated with the syndrome. This case highlights the importance of good communication between medical and paramedical departments, as well as the family of the patient, particularly in pediatric patients. Also, we see the importance of multidisciplinary care and of looking ahead to anticipate probable complications and comorbidities that are likely to occur. As a result of this, as well as full-time care at home by her mother, the physical health of the patient is well maintained.
